# Knowledge, attitude and practices among parents regarding food poisoning: a cross-sectional study from Palestine

**DOI:** 10.1186/s12889-019-6955-2

**Published:** 2019-05-16

**Authors:** Sa’ed Zyoud, Jawad Shalabi, Kathem Imran, Lina Ayaseh, Nawras Radwany, Ruba Salameh, Zain Sa’dalden, Labib Sharif, Waleed Sweileh, Rahmat Awang, Samah Al-Jabi

**Affiliations:** 1 Poison Control and Drug Information Center (PCDIC), College of Medicine and Health Sciences, An-Najah National University, Nablus, 44839 Palestine; 2 Department of Clinical and Community Pharmacy, College of Medicine and Health Sciences, An-Najah National University, Nablus, 44839 Palestine; 3 PharmD program, College of Medicine and Health Sciences, An-Najah National University, Nablus, 44839 Palestine; 4 Department of Veterinary Pathology and Public Health, Faculty of Veterinary Medicine, Jordan University of Science and Technology, Irbid, 22110 Jordan; 5 Department of Pharmacology and Toxicology, College of Medicine and Health Sciences, An-Najah National University, Nablus, 44839 Palestine; 6 WHO Collaborating Centre for Drug Information, National Poison Centre, Universiti Sains Malaysia (USM), 11800 Penang, Malaysia

**Keywords:** Parents, Children, Food poisoning, Food safety, Palestine

## Abstract

**Background:**

Food serves as a vehicle for many pathogenic and toxigenic agents that cause food-borne diseases. Knowledge, attitude, and practices are key factors in reducing the incidence of food-borne diseases in food service areas. The main objective of this study was to evaluate knowledge, attitude, and practices related to food poisoning among parents of children in Nablus, Palestine.

**Methods:**

A cross-sectional study was conducted in primary healthcare centers in Nablus district from May to July 2015. Data were collected using structured questionnaire interviews with parents to collect information on food safety knowledge, attitudes, and practices, alongside sociodemographic characteristics.

**Results:**

Four-hundred and twelve parents were interviewed, 92.7% were mothers. The median knowledge score was 12.0 with an interquartile range (IQR) of 11.0–14.0. The median attitude score was 11.0 with IQR of 10.0–13.0, while the median practice score was 18.0 with IQR of 16.0–19.0. Significant modest positive correlations were found between respondents’ knowledge and attitude scores regarding food poisoning (*r* = 0.24, *p* < 0.001), knowledge and practice scores regarding food poisoning (*r* = 0.23, *p* < 0.001), and attitude and practice scores regarding food poisoning (*r* = 0.30, *p* < 0.001). Respondents with a higher education level and who live in a city were the only factors significantly associated with higher knowledge scores (*p* < 0.05). Attitude improved as educational level increased (*p* < 0.05) and income level increased (*p* < 0.05). Those of female gender and employed were statistically significantly associated with higher satisfactory hygienic practices in relation to the prevention of food poisoning (*p* < 0.05).

**Conclusions:**

Knowledge, attitude, and practices regarding food poisoning prevention are associated with each other and are affected by a complex interplay between socio-economic variables. The study highlights the need for health education programmes and general awareness campaigns that intend not only to enhance knowledge but also promote parents to practice food safety measures strictly and further strengthen their awareness level.

**Electronic supplementary material:**

The online version of this article (10.1186/s12889-019-6955-2) contains supplementary material, which is available to authorized users.

## Background

Food serves as a vehicle for many pathogenic and toxigenic agents that cause what are known as food-borne diseases or food poisoning [1]. In recent decades, food poisoning has become a growing public health problem worldwide, in both developed and developing countries [2–6]. It is defined as a variety of illnesses acquired by consumption of contaminated foods or water, with a variety of causes ranging from infective organisms (bacteria and viruses), poisonous chemicals, radioactive substances and other harmful substances leading to more than 250 different food-borne diseases (ranging from diarrhoea to cancers) [7]. Food safety refers to the processes of handling, preparing and storing of food in ways that prevent contamination by toxic chemicals or pathogenic microbes resulting in food-borne illness [8].

Symptoms of toxigenic food poisoning mostly appear within 24 h after ingestion of contaminated food while foodborne infections may not appear until 2–3 days later. Symptoms can include nausea, vomiting, diarrhea, abdominal pain (which is severe in inflammatory processes), headache and fever. Life-threatening neurologic, hepatic and renal syndromes may occur several days after intestinal symptoms, and may cause permanent disability or death depending upon which microbe is ingested [1, 9].

In developing countries, many poisoning cases are due to the consumption of unhygienic foods, pesticide residues in foods or water, and inappropriate food storage conditions. However, food poisoning is not a distinctive phenomenon of these countries only, as it also involves developed countries, due to the increasing demand for low-priced food and lack of ability to provide optimal care under hygienic conditions while preparing and storing food [5, 10].

The incidence of food-borne illness depends on the hygienic measures implicated in food production and storage, but they could be ineffective if consumers have poor hygienic practices and food handling approaches [11]. Approximately 50% of food-borne illness cases are related to improper storage or reheating, with 45% associated with inappropriate food storage and 39% with cross contamination [12]. Knowledge, attitude and practice are key factors in reducing the incidence of food-borne diseases in foodservice areas [13]. They are also influenced by various factors like gender (females have a higher level of information than males), age (people younger than 35 years of age need extra food safety education), income level and cultural factors [14, 15]. Therefore, evaluation of the baseline of such data (before any food safety education material can be prepared for their use) is an essential first step to inform the development of effective and relevant health education programmers to ensure that food handlers are knowledgeable about and experienced in food safety [16, 17].

To the best of our knowledge, there are few studies conducted in Palestine regarding food poisoning or food-borne illnesses [18–22]. Therefore, the main objective of this study was to evaluate knowledge, attitude and practices related to food poisoning among parents in Nablus district, Palestine. However, regarding parents’ practices and knowledge regarding food poisoning in Palestine, our study may be the first one conducted in Palestine.

## Methods

### Study design and sampling strategy

In the current study, a descriptive cross-sectional method was applied. The study was conducted in four primary healthcare centres in Nablus, Palestine: Al-Makhfeyah; Ras-Alain; Al-Wosta; and Balata. Parents attending the centers with children aged less than 6 years were interviewed and their answers were transcribed.

A non-probability convenience sampling technique was used. A sample of 104 parents was selected from each primary healthcare centre. Inclusion criteria included parents 18 years old or older and gave consent to take part in the study. Exclusion criteria included parents who were not resident in the study area or had not given birth or who had children over 6 years or who had difficulty in communication.

### Data collection form

A questionnaire in the native Arabic language adapted from a previous study [23] was used. The questionnaire consisted of four sections (see Additional file 1):

- The first section included demographic information such as participants’ age, gender, educational level, employment status, residency, income level, number of family members including children, their ages, food preparation, and number of meals consumed away from home.
- The second section consisted of 15 questions about food poisoning knowledge including general statements about food poisoning causes.
- The third section consisted of 15 questions about food poisoning attitude including statements about eating raw food and washing fruits and vegetables.
- In the fourth section, consisted of 20 questions about food poisoning practices regarding eating, drinking and washing hands.

In the knowledge and attitude section, the questionnaire provided five choices ranging from ‘strongly agree’ to ‘strongly disagree’. In the practice section, the respondents had five options: ‘always yes’; ‘most of the time’; ‘sometimes’; ‘rarely’; and ‘always no’. The questionnaire was tested with a pilot sample (*N* = 30) before conducting the study and no changes were recommended. Permission to use this instrument to measure parents’ knowledge, attitude and practices regarding food poisoning in this study was obtained from the developers of the questionnaire [23].

### Data collection procedure

Clinical pharmacists, who were well-trained on collecting data for the knowledge, attitude and practices questionnaire, carried out data collection. Parents were asked to complete the questionnaire during a face-to-face interview. Each questionnaire took 10–15 min to administer. Statements in the questionnaire were explained when necessary. A verbal consent form, explaining the purpose of the research and assuring confidentiality was read to participants. The participants had the right to participate or not. The data were collected on weekdays only. The collection process occurred from May to July 2015.

### Ethical consideration

The Institutional Review Boards (IRB) of An-Najah National University authorized all aspects of the study protocol before the initiation of the study and approval to conduct the study was obtained from the Ministry of Health. Verbal consent was also obtained from all parents before participation.

### Data statistical analysis

All analyses were performed using IBM SPSS statistics, version 20.0. Descriptive statistics were used to describe the sample. The responses were analyzed as correct or incorrect answers (see Additional file 2). Each correct answer was given one point while each incorrect answer was given zero point. Regarding knowledge, the five choices that ranged from ‘strongly agree’ to ‘strongly disagree’ were given values of 4 to 0, respectively. A correct answer indicated good knowledge, whereas an incorrect answer indicated poor knowledge. For attitude, this section included a set of negative statements (1–11) and the other four statements were positive (12–15). Negative statement choices had a value of 0 for ‘strongly agree’ and 4 for ‘strongly disagree’ and for positive statements it was the opposite (4 for ‘strongly agree’ and 0 for ‘strongly disagree’). A correct answer indicated a good attitude and an incorrect answer indicated a poor attitude. The practice section included six positive questions (1–6) with an answer choice value of 4 for ‘always yes’ and 0 for ‘always no’. The remaining questions (7–20) were negative with an answer choice value of 0 for ‘always yes’ and 4 for ‘always no’. A correct answer was indicated hygienic practice and a negative answer indicated unhygienic practice. Responses were consolidated into dichotomous (binary) variables for analysis. Previous studies had shown that consolidating a Likert scale to binary formats does not affect the results [24, 25]. Data were presented as mean ± standard deviation (SD) or median and interquartile range (IQR) for numerical variables, and frequencies with percentages for nominal variables. All scores were tested for normality using the Kolmogorov–Smirnov test. Because the normality of all scores were not met, the Mann-Whitney U test and the Kruskal-Wallis H test were performed. The Pearson correlation coefficient was calculated to examine a possible correlation between continuous variables (knowledge, attitude and practices scores). In all analyses, a significance level of 5% was used.

## Results

### Sociodemographic data

Demographic information for the sample is presented in Table 1. Four-hundred and twelve questionnaires were collected from the primary care centres. Respondents were mostly females (92.7%). The mean age of respondents was 29.62 years (SD ± 6.380) and the median was 29.0 years with an interquartile range of 25.0–34.0. For educational level, the highest percentage was for high educational level followed by secondary level (40.8 and 38.8%, respectively). More than half of the respondents (66.9%) were living in a city and had an average income level of 500–1000 Jordanian Dinar (1 Jordanian Dinar (JD) equals 1.41 US Dollar); (59.2%). The majority was unemployed (78.9%) and reported that the mother is responsible for food preparation (96.3%).

**Table 1:** Demographic data of the sample (*n* = 412)

Variable	Number (%) or median [interquartile range]
Age group	
18–20	24 (5.8)
21–30	224 (54.4)
31–40	137 (33.3)
41–50	27 (6.5)
Gender	
Male	30 (7.3)
Female	382 (92.7)
Educational level	
Illiterate/Primary	84 (20.4)
Secondary	160 (38.8)
High	168 (40.8)
Employment status	
Working	87 (21.1)
Not working	325 (78.9)
Residency	
City	276 (66.9)
Village	34 (8.3)
Palestinian refugee camps	102 (24.8)
Income level of the family ^a^	
Low (less than 500JD)	108 (26.2)
Average (500-1000JD)	244 (59.2)
High (1001- >3000JD)	60 (14.6)
Family members number	
2–5	250 (60.7)
> 5	162 (39.3)
Number of children	
1–3	256 (62.1)
4–6	140 (34.0)
> 6	16 (3.9)
Children ages	
0–6 only	213 (51.7)
0–6 and > 6	199 (48.3)
Food preparing ^b^	
Mother	395 (96.3)
Father	15 (3.7)
Number of meals consumed away from home	
Never	185 (44.9)
1–3 times/month	185 (44.9)
1–2 times/week	32 (7.8)
> 2 times/week	10 (2.4)
Knowledge score	12.0 [11.0–14.0]
Attitude score	11.0[10.0–13.0]
Practice score	18.0 [16.0–19.0]

^a^1 Jordanian Dinar (JD) equals 1.41 US Dollar

^b^ There are two samples not included

### Knowledge about food poisoning

The total knowledge score was 15. The mean knowledge score was 12.0 (SD = 2.2) and the median was 12.0 with an interquartile range of 11.0–14.0. Distribution of responses to each knowledge question is shown in Additional file 2: Table S1. As shown in Additional file 2: Table S1, 88.4% of the respondents agreed that pathogenic microbes cause food poisoning. When the respondents were asked about the highly risky foods for food poisoning, 93.2% of the respondents correctly agreed that eating raw or half-cooked meat is highly risky for food poisoning, 91.5% agreed that eating raw unwashed vegetables is highly risky for food poisoning and 87.1% agreed that drinking raw milk is highly risky for food poisoning. Moreover, 84.7, 82.8, 78.3, 74.3 and 70.9% of the respondents agreed correctly that raw white cheese, unwashed not pealed fruits, uncovered leftover cooked food, untreated surface and rainwater, and raw eggs, respectively, were risky food.

The majority of respondents agreed that well cooked food is free from microbes that cause food poisoning (91.3%) and that keeping food at refrigerator will slow down the microbial growth and multiplication, so, prevent food poisoning (90.2%). Approximately two thirds of the respondents agreed that there is no risk of food poisoning from eating leftover cooked food kept in refrigerator for 2-3 days. Eighty three percent of the respondents agreed that unhygienic practice of food handlers could be the source of microbial contamination of the food, which causes food poisoning. On the other hand, only 49.0% of the respondents correctly agreed that some toxins produced by microbes and cause food poisoning are resistant to heating temperature of food. Detailed responses are shown in Additional file 2: Table S1 and S2.

The Kruskal-Wallis test was used to analyse the association between sociodemographic factors and knowledge. According to that, both educational level and residence area show a significant association with knowledge (*p* < 0.05) (Table 2). On the other hand, there was no significant association between age and gender with knowledge. Respondents with a high level of education reported good knowledge (median = 13.0), more so than others with secondary or primary level (median = 12.0). As the educational level increased, knowledge increased (*p* < 0.05). Respondents who live in a city or village reported good knowledge (median = 13.0), more so than those living in a camp (median = 11.0). Knowledge increased as residency situation improved (*p* < 0.05).

**Table 2:** Association between socio-demographic factors and knowledge of food poisoning

Variable ^#^	Number (%) *N* = 412	Knowledge score Median [interquartile range] 12.0 [11.0–14.0]
Age group ^a^		
18–20	24 (5.8)	11.5[9.3–13.0]
21–30	224 (54.4)	12.0[11.0–14.0]
31–40	137 (33.3)	13.0[11.0–14.0]
41–50	27 (6.5)	12.0[10.0–14.0]
Gender ^b^		
Male	30 (7.3)	12.0[9.8–14.0]
Female	382 (92.7)	12.0[11.0–14.0]
Educational level ^*, a^		
Illiterate/Primary	84 (20.4)	12.0[10.0–13.0]
Secondary	160 (38.8)	12.0[11.0–13.0]
University	168 (40.8)	13.0[11.0–14.0]
Employment status ^b^		
Working	87 (21.1)	12.0[11.0–14.0]
Not working	325 (78.9)	12.0[11.0–14.0]
Residency ^*, a^		
City	276 (66.9)	13.0[11.0–14.0]
Village	34 (8.3)	13.0[11.0–14.0]
Palestinian refugee camps	102 (24.8)	11.0[10.0–12.0]
Income level of the family ^a, c^		
Low (less than 500JD)	108 (26.2)	12.0[11.0–13.8]
Average (500-1000JD)	244 (59.2)	12.0[11.0–14.0]
High (1001- >3000JD)	60 (14.6)	12.0[10.0–13.0]
Family members number ^b^		
2–5	250 (60.7)	12.0[11.0–14.0]
> 5	162 (39.3)	12.0[11.0–14.0]
Number of children ^a^		
1–3	256 (62.1)	12.0[11.0–14.0]
4–6	140 (34.0)	12.0[11.0–14.0]
> 6	16 (3.9)	12.5[10.5–13.8]
Children ages ^b^		
0–6 only	213 (51.7)	12.0[11.0–14.0]
0–6 and > 6	199 (48.3)	12.0[11.0–14.0]
Food preparing ^d^		
Mother	395 (96.3)	12.0[11.0–14.0]
Father	15 (3.7)	12.0[10.0–13.0]
Number of meals consumed away from home ^a^		
Never	185 (44.9)	12.0[11.0–13.0]
1–3 times/month	185 (44.9)	13.0[11.0–14.0]
1–2 times/week	32 (7.8)	12.0[10.0–14.0]
> 2 times/week	10 (2.4)	12.5[10.3–14.0]

^#^For each variable, a value with the asterisk (^*^) signifies *P* < 0.05 and the absence of asterisk indicates otherwise

^a^*p*-value is calculated using Kruskal-Wallis test

^b^*p*-value is calculated using Mann-Whitney U test

^c^1 Jordanian Dinar (JD) equals 1.41 US Dollar

^d^There are two samples not included

### Attitude regarding food poisoning

The total attitude score was 15, while the mean attitude score was 11.1 (SD = 2.5) and the median was 11.0 with an interquartile range of 10.0–13.0. Distribution of responses to each attitude question is shown in Additional file 2: Table S3. As shown in Additional file 2: Table S3, the majority of the respondents reported that washing hands with soap before preparing (96.4%) and eating food (96.1%) along with thorough washing of vegetables and fruits (95.6%) are necessary to prevent food poisoning. Two thirds of the respondents correctly agreed that food handlers without clinical symptoms could contaminate food with pathogenic microbes, which cause food poisoning. Regarding raw milk, 82.0% of the respondents correctly disagreed that there is no risk of disease from drinking raw goat or cow milk right after milking, 76.7% correctly disagreed that raw milk is more healthy and nutritious than pasteurized or boiled milk and 71.4% disagreed there is no risk of disease from drinking the milk of the camel right after milking. Regarding raw eggs, 68.4% of respondents correctly disagreed that raw eggs are more healthy and nutritious than cooked ones, while 36.2% wrongly agreed that there is no risk of disease from drinking raw eggs. A great percentage of the respondents correctly disagreed the statement that there is no risk of disease from eating raw meat of young animals. Regarding vegetables and fruits, 82.8% of the respondents disagreed that wiping vegetables or fruits make them safe to be eaten and 79.9% disagreed the statement that there is no risk of disease from eating unwashed vegetables and herbs picked up directly from the plant. On the other hand, about two thirds of the respondents wrongly agreed that baby feces are free from pathogenic microbes if he/she is not sick. About forty-six percentage of the respondents wrongly agreed that rainwater collected in reservoir is safe to drink without any treatment and 43.7% agreed that there is no risk of disease from eating cooked food kept at room temperature for 1 day if covered. Detailed responses are shown in Additional file 2: Table S3 and S4.

Table 3 shows the significant association between attitude and two of the demographics: educational level and income level (Kruskal-Wallis test, *p* < 0.05). Age, gender, employment status and residency did not show a significant association with attitude. Respondents with a high educational level reported a good attitude (median = 12.0), more than those with lower levels (median = 11.0). As the educational level increased, attitude was improved (*p* < 0.05). Respondents who have high-income levels (i.e. 1001–2999 JD (1 JD equals 1.41 US Dollar)) reported a good attitude (median = 12.0), more than those having a lower one (median = 11.0). As income level increased, attitude improved (*p* < 0.05).

**Table 3:** Association between socio-demographic factors and attitude towards food poisoning

Variable ^#^	Number (%) *N* = 412	Attitude score Median [interquartile range] 11.0[10.0–13.0]
Age group ^a^		
18–20	24 (5.8)	11.0[8.0–12.8]
21–30	224 (54.4)	12.0[10.0–13.0]
31–40	137 (33.3)	11.0[9.0–13.0]
41–50	27 (6.5)	12.0[11.0–13.0]
Gender ^b^		
Male	30 (7.3)	11.0[8.0–12.3]
Female	382 (92.7)	11.0[10.0–13.0]
Educational level ^*, a^		
Illiterate/Primary	84 (20.4)	11.0[10.0–13.0]
Secondary	160 (38.8)	11.0[9.0–13.0]
High	168 (40.8)	12.0[10.0–13.0]
Employment status ^b^		
Working	87 (21.1)	12.0[10.0–13.0]
Not working	325 (78.9)	11.0[10.0–13.0]
Residency ^a^		
City	276 (66.9)	12.0[10.0–13.0]
Village	34 (8.3)	11.0[10.0–13.0]
Palestinian refugee camps	102 (24.8)	11.0[9.0–13.0]
Income level of the family^*, a, c^		
Low (less than 500JD)	108 (26.2)	11.0[10.0–13.0]
Average (500-1000JD)	244 (59.2)	11.0[10.0–13.0]
High (1001- >3000JD)	60 (14.6)	12.0[11.0–14.0]
Family members number ^b^		
2–5	250 (60.7)	12.0[10.0–13.0]
> 5	162 (39.3)	11.0[10.0–13.0]
Number of children ^a^		
1–3	256 (62.1)	11.0[10.0–13.0]
4–6	140 (34.0)	11.0[10.0–13.0]
> 6	16 (3.9)	11.0[9.0–13.0]
Children ages		
0–6 only	213 (51.7)	12.0[10.0–13.0]
0–6 and > 6	199 (48.3)	11.0[10.0–13.0]
Food preparing ^b, d^		
Mother	395 (96.3)	11.0[10.0–13.0]
Father	15 (3.7)	11.0[10.0–13.0]
Number of meals consumed away from home ^a^		
Never	185 (44.9)	11.0[10.0–13.0]
1–3 times/month	185 (44.9)	12.0[10.0–13.0]
1–2 times/week	32 (7.8)	12.0[10.0–13.8]
> 2 times/week	10 (2.4)	10.5[9.5–12.5]

^#^For each variable, a value with the asterisk (^*^) signifies *P* < 0.05 and the absence of asterisk indicates otherwise

^a^*p*-value is calculated using Kruskal-Wallis test

^b^*p*-value is calculated using Mann-Whitney U test

^c^1 Jordanian Dinar (JD) equals 1.41 US Dollar

^d^There are two samples not included

### Practices regarding food safety

The total practice score was 20. The mean practice score was 17.3 (SD = 2.6) and the median was 18.0 with an interquartile range of 16.0–19.0. Distribution of responses to each practices question is shown in Additional file 2: Table S5. As shown in Additional file 2: Table S5, parents’ responses suggest that 95.9% of the respondents wash their hands with soap and water before both eating and preparing food, after contact with animals (98.3%) and after using the toilet (99.3%). About 98% of the respondents wash fresh vegetables and fruits before eating and 72.3% wash their hands with water and soap after handling raw unwashed vegetables. On the other hand, more than one third of the respondents may eat fresh vegetables and fruits after just wiping it (35.9%), without washing it (31.8%) or pick up it from the plants during a field trip and eat it without washing (34.7%). The majority of the respondents do not eat raw eggs, but about one quarter of them may eat half-cooked eggs (i.e. egg yolk is soft) (24.8%). Over 90 % of the respondents do not eat raw or half-cooked meat (96.1 and 94.2%, respectively) and do not drink raw cow, goat (94.2%), or camel milk (94.4%). Although that, about 20 % of the respondents may eat raw white cheese prepared from raw un-pasteurized milk. When the respondents were asked about cooked food left at room temperature for over 6 h without sufficient heating, only 28.2% said that they might eat it. A high percentage of respondents don’t eat from a restaurant or cafeteria looks not clean (96.4%), don’t drink from rainwater collected without any treatment (83.3%) and don’t eat food, like meat and rice and soup, by hand from a bowl shared by several people (91.7%). Detailed responses are shown in Additional file 2: Table S5 and S6.

The results as shown in Table 4 indicate that gender and employment status were significantly associated with practice towards food poisoning with *p* < 0.05 (Mann-Whitney U test) and residency was significantly associated with it (Kruskal-Wallis test, *p* < 0.05). Other demographics did not show any significant association with practice towards food poisoning like age, educational level and income level. Female participants reported hygienic practice (median = 18.0) more than male participants (median = 16.0), so, hygienic practice towards food poisoning is higher in females than males (*p* < 0.05). Hygienic practice towards food poisoning is higher among non-working consumers than working ones (*p* < 0.05). The medians for both were 18.0 and 17.0, respectively. Respondents who live in a village reported hygienic practice towards food poisoning (median = 19.0), more than those living in a city (median = 18.0). Those living in a camp reported the lowest hygienic practice (median = 16.0). Living situation improvement is not necessary to increase hygienic practice towards food poisoning (*p* < 0.05).

**Table 4:** Association between socio-demographic factors and practice towards food poisoning

Variable ^#^	Number (%) *N* = 412	Practice score Median [interquartile range] 18.0 [16.0–19.0]
Age group ^a^		
18–20	24 (5.8)	18.0[15.3–19.0]
21–30	224 (54.4)	18.0[16.0–19.0]
31–40	137 (33.3)	18.0[16.0–19.0]
41–50	27 (6.5)	17.0[14.0–19.0]
Gender ^*, b^		
Male	30 (7.3)	16.0[14.0–18.0]
Female	382 (92.7)	18.0[16.0–19.0]
Educational level ^a^		
Illiterate/Primary	84 (20.4)	18.0[15.0–19.0]
Secondary	160 (38.8)	18.0[16.0–19.0]
High	168 (40.8)	18.0[16.0–19.0]
Employment status ^*, b^		
Working	87 (21.1)	17.0[15.0–19.0]
Not working	325 (78.9)	18.0[16.0–19.0]
Residency ^*, a^		
City	276 (66.9)	18.0[17.0–19.0]
Village	34 (8.3)	19.0[16.8–20.0]
Palestinian refugee camps	102 (24.8)	16.0[15.0–17.0]
Income level of the family ^a, c^		
Low (less than 500JD)	108 (26.2)	18.0[16.0–19.0]
Average (500-1000JD)	244 (59.2)	18.0[16.0–19.0]
High (1001- >3000JD)	60 (14.6)	17.0[15.3–18.8]
Family members number ^b^		
2–5	250 (60.7)	18.0[16.0–19.0]
> 5	162 (39.3)	18.0[16.0–19.0]
Number of children ^a^		
1–3	256 (62.1)	18.0[16.0–19.0]
4–6	140 (34.0)	18.0[16.0–19.0]
> 6	16 (3.9)	17.0[12.5–19.0]
Children ages ^b^		
0–6 only	213 (51.7)	18.0[16.0–19.0]
0–6 and > 6	199 (48.3)	18.0[16.0–19.0]
Food preparing ^b, d^		
Mother	395 (96.3)	18.0[16.0–19.0]
Father	15 (3.7)	19.0[15.0–20.0]
Number of meals consumed away from home ^a^		
Never	185 (44.9)	18.0[16.0–19.0]
1–3 times/month	185 (44.9)	18.0[16.0–19.0]
1–2 times/week	32 (7.8)	19.0[17.0–19.8]
> 2 times/week	10 (2.4)	17.5[13.3–20.0]

^#^For each variable, a value with the asterisk (^*^) signifies *P* < 0.05 and the absence of asterisk indicates otherwise

^a^*p*-value is calculated using Kruskal-Wallis test

^b^*p*-value is calculated using Mann-Whitney U test

^c^1 Jordanian Dinar (JD) equals 1.41 US Dollar

^d^There are two samples not included

### The correlations between knowledge, attitude and practice scores regarding food poisoning

A significant modest positive correlation was shown between respondents’ knowledge and attitude scores regarding food poisoning (*r* = 0.240, *p* < 0.001). The results mean that respondents who had good knowledge were more likely to have a good attitude towards food poisoning. A significant modest positive correlation was demonstrated between respondents’ knowledge and practice scores regarding food poisoning (*r* = 0.227, *p* < 0.001). Taken together, these results indicate that respondents who had good knowledge were more likely to have hygienic practices towards food poisoning. There was a significant modest positive correlation between respondents’ attitude and practice scores regarding food poisoning (*r* = 0.303, *p* < 0.001), which means that respondents who had a good attitude were more likely to have hygienic practice towards food poisoning.

## Discussion

This study was designed to assess and evaluate the level of knowledge, attitude and practices among Palestinian parents regarding food poisoning and to determine if they have acceptable levels of them. As mentioned in the literature review, there are no studies undertaken to discuss parents’ knowledge, attitude and practices regarding food poisoning in Palestine.

The most obvious note to emerge from the analysis is that the sample has a bias to female participation with *n* = 382 female participants and *n* = 30 male participants. Additional factors such as parity, rural/urban status, household wealth status, and spouses’ education level would help to get a more complete view of possible predictive factors related to knowledge, attitude and practices towards food poisoning. However, in Middle Eastern Arab cultures, the female carries the responsibility for family care as a wife, from cleaning and arranging the house to preparing and cooking the food for all members of the family [26, 27]. In addition, the female, as a mother, takes care of her children, and being a daughter, she will help her mother. Because of this responsibility, being the housekeeper generally and especially the main food preparer, she always seeks to protect her family members and does not want to cause them any suffering. Food preparers believe that any food prepared outside the home (i.e. in restaurants, cafeterias, hotels... etc.) will make them more susceptible to food poisoning and do not think that food prepared in the home may contribute to food poisoning [11, 28–30]. A study that was conducted in the West Indies revealed that consumers think that 20% of food poisoning occurs in the home [28]. Although Day [31] found that the percentage of cases arising from food prepared at home may be underestimated in statistics for many reasons. Borneff et al. [32] reported that food-borne illness caused by food prepared at home is three times that caused by food prepared in cafeterias. The WHO reported that 40% of food-borne outbreaks occur at home [33]. Alsayeqh [34] found that insufficient cooking, unsatisfactory storage of food, cross contamination, and unsafe food sources are risks for food poisoning, Williamson et al. [35] found that 25% of food poisonings are due to inappropriate food handling and preparation, while Redmond & Griffith [16] estimated that 50–86% of reported food-borne outbreaks are associated with the home.

Several studies have shown that there are many variables that affect food safety knowledge, attitude, behaviours, perception and practice. Age, gender, education level, socio-economic status and employment status were the most important variables [15, 17, 36, 37]. Food safety knowledge has been found to increase with age and length of practice, and females reported higher scores than males [38]. Bruhn and Schutz [39], Byrd-Bredbenner et al. [40] and Sudershan et al. [30] found that females have more information about food safety and appropriate food handling methods than males. Many studies that were conducted to examine the food safety knowledge among young adults further support the previous research [23, 41].

On the other hand, Badrie et al. [28] reported that gender has no effect in the responses of consumers, while Unusan [42] found that males scored higher than females in food safety knowledge. Many studies found that very young or old adults, men, those of low educational and income levels have higher risky behaviours than younger people [5, 43]. A possible explanation for low knowledge in young adults may be due to a lack of home economics courses in secondary schools that teach food safety [41, 44]. Consumers with college education and those that work in food or nutrition related jobs reported higher knowledge scores than those with a high school degree and who had never worked in this area because of the training classes required of the employee [11]. Patil et al. [26] found that consumers with college education reported less safe practices than those without higher education, although they reported higher knowledge scores. Risky behaviours increase with increasing socio-economic status [45]. This inconsistency may be because safe food handling and consumption practices are developed by preparation experience in food preparing [46]. In other words, consumers living in higher socio-economic levels do not prepare food as often as those from lower levels.

In this study, the results seem to be consistent with other research which found that there was a significant association between educational level and knowledge score. Respondents with a high level of education reported higher knowledge scores than those with a lower level. In addition, respondents who live in a city or village reported higher scores than those living in camps. Another important finding was that age affects knowledge score, but the association was not significant. Surprisingly, gender affected neither knowledge nor attitude but significantly affected practice. This finding further supports the idea that females are more informed about appropriate food handling behaviours. Respondents with a high education level reported a good attitude compared to those with a lower level. Income level was also found to affect attitude score in that respondents with an income level of 1001–2999 JD (1 JD equals 1.41 US Dollar) scored higher than others. These results support the ideas [29, 44, 47] mentioned previously. In addition to gender, residency and employment status show a significant association with practice. Respondents who live in a village had greater hygienic practice than those living in a city, while those living in a camp reported the lowest hygienic practice. Working respondents scored lower than those who are not working. A possible explanation for this result may be the lack of adequate experience in food preparation between working respondents compared to those not working. These results support the findings of Issa et al. [22] that camp residents lack hygiene awareness and knowledge regarding food handling.

Redmond and Griffith [16] and Yarrow et al. [48] said that knowledge does not always participate in developing a positive attitude and behaviours, and Hogue et al. [49] reported that consumers have knowledge about food safety, but their knowledge is not always reflected in their food handling behaviours. In contrast to earlier findings, this study showed that there is a significant modest positive correlation between knowledge and both attitude and practice. In addition, there was a significant modest positive correlation between attitude and practice. As long as the consumer had good knowledge, he/she was more likely to have a good attitude and hygienic practice and as long as he/she had a good attitude, he/she had hygienic practice. These findings support those of Al-Shabib and his colleagues [50]. After analysing the data, it was somewhat encouraging and pleasing that 96.12, 90.77 and 98.30% of the respondents had good knowledge, a good attitude and hygienic practice, respectively.

The present results are significant in that they could be taken as a baseline for educating people about proper practice to reduce food poisoning. However, there are still many questions about this topic (i.e. food poisoning knowledge, attitude and practice in Palestine) and more research is needed to form a correct and clear view.

## Strengths and limitations

The major strength of the present study is the relatively large sample size. In addition, a key strength of this study is that it is the first one in Palestine conducted to evaluate knowledge, attitude and practices level regarding food poisoning for parents. The major limitation of this study is that the data were collected by face-to-face interview, so, the respondents may answer in a manner that makes them look well informed, yet it does not give the real answers, thus we only measured and compared the reported practice and did not know if consumers actually did what they report doing. Being limited to Nablus, this study lacks representativeness of all Palestinian parents and represents only those in Nablus district. Another limitation that may affect the generalizability of the study is that the majority of the respondents were female and only 7.3% male. We also found that this analysis involved cross-sectional data so we are unable to determine the direction of the relationship between parents’ factors and levels of knowledge, attitude and practices towards food poisoning.

## Conclusions

The main goal of the current study is to evaluate knowledge, attitude and practices regarding food poisoning among Palestinian parents in Nablus. This study shows that the respondents generally have good knowledge, a good attitude and hygienic practices (96.12, 90.77 and 98.30%, respectively). The study also found a significant positive correlation between knowledge, attitude and practice. Highly educated respondents and those who live in cities or villages have better knowledge than others.

Moreover, highly educated respondents and those with high-income levels have better attitudes. Unemployed females, as they have higher chance to prepare food at home, living in villages are more likely to have hygienic practices. In general, it seems that there is an emerging need for appropriate solutions. These findings suggest significant implications to be considered as a baseline for further investigations, and for building and applying awareness campaigns for educating the public on the importance of basic food safety. Educational programmes or campaigns should start with parental awareness regarding the direct hazards to their family health from food poisoning, as by realising their susceptibility to food poisoning, their motivation to change becomes stronger and beneficial, as parents are more likely to change their behaviours as long as it benefits their children.

## Additional file

Additional file 1: Study questionnaires. This is the final English version of the questionnaire that was used to obtain data that helps to evaluate knowledge, attitude, and practices related to food poisoning among parents of children in Nablus, Palestine. (DOCX 27 kb)

Additional file 2: **Table S1**. Parents’ knowledge responses with correct answers. **Table S2.** Distribution of responses to each knowledge question with a five-point Likert scale ranked from 1 to 5 (Strongly disagree, disagree, not sure, agree, and strongly agree). **Table S3.** Parents’ attitude responses with correct answers. **Table S4.** Distribution of responses to each attitude question with a five-point Likert scale ranked from 1 to 5 (Strongly disagree, disagree, not sure, agree, and strongly agree). **Table S5.** Parents’ practice responses with correct answers. **Table S6.** Distribution of responses to each practice question with a five-point Likert scale ranked from 1 to 5 (Always yes, most of the time, sometimes, rarely, always no). (DOCX 27 kb)

## Abbreviations

IQR: Interquartile range
IRB: Institutional Review Boards
SD: Standard deviation
