# Supplementary material for: Knowledge, attitude and practices among parents regarding food poisoning: a cross-sectional study from Palestine
Source: BMC Public Health. 2019 May 16;19:586. doi: 10.1186/s12889-019-6955-2 (PMC6524328; doi:10.1186/s12889-019-6955-2)
Supplement: Supplementary file 1 — Study questionnaires. This is the final English version of the questionnaire that was used to obtain data that helps to evaluate knowledge, attitude, and practices related to food poisoning among parents of children in Nablus, Palestine. (DOCX 27 kb) [file 12889_2019_6955_MOESM1_ESM.docx]

**Additional file 1: Study questionnaires.** This is the final English version of the questionnaire that was used to obtain data that helps to evaluate knowledge, attitude, and practices related to food poisoning among parents of children in Nablus, Palestine.

**First section: Demographic data.**

**Age**……...

**Gender:** □ Male □ Female

**Educational level for mother:** □ Illiterate

□ Primary

□Secondary

□ High

**Educational level for father:** □ Illiterate

□ Primary

□Secondary

□ High

**Employment status:** □ Working

□ Not working

**Residency:** □ City

□ Village

□ Palestinian refugee camp

**Income level of the family:** □Low (less than 500JD)

□ Average (500-1000JD)

□ High (1001- >3000JD)

**Family members number**…….

**Number of children**……………

**Children ages:** □0-6 ………... □More than 6………………

**Who cooks at home:** □ Mother

□ Father

□ Other

**Number of meals consumed away from home:** □ Never

□ 1-3 times/month

□1-2 times/week

□ >2 times/week

**Second section: Food poisoning knowledge.**

| **#** | **Statement #*** | **Strongly**  **agree** | **Agree** | **Uncertain** | **Disagree** | **Strongly**  **disagree** |
| --- | --- | --- | --- | --- | --- | --- |
| 1 | Food poisoning is caused by pathogenic microbes. |  |  |  |  |  |
| 2 | Some toxins produced by microbes and cause food poisoning are resistant to heating temperature of food. |  |  |  |  |  |
| 3 | Drinking raw milk is highly risky for food poisoning. |  |  |  |  |  |
| 4 | Eating raw eggs is highly risky for food poisoning. |  |  |  |  |  |
| 5 | Eating raw or half-cooked meat is highly risky for food poisoning. |  |  |  |  |  |
| 6 | Eating raw unwashed vegetables is highly risky for food poisoning. |  |  |  |  |  |
| 7 | Eating unwashed and not pealed fruits is highly risky for food poisoning. |  |  |  |  |  |
| 8 | Food handlers with unhygienic practice could be the source of microbial contamination of the food which causes food poisoning |  |  |  |  |  |
| 9 | Well cooked food is free from microbes which cause food poisoning. |  |  |  |  |  |
| 10 | Eating uncovered leftover cooked food, kept at room temperature for 12–24 h, is at high risk to cause food poisoning. |  |  |  |  |  |
| 11 | Raw white cheese processed from raw milk has a high risk of food poisoning. |  |  |  |  |  |
| 12 | Pasteurized milk can be drunk directly with no risk of food poisoning. |  |  |  |  |  |
| 13 | Keeping food at refrigerator temperature will slow down the microbial growth and multiplication, thus prevent food spoilage and food poisoning. |  |  |  |  |  |
| 14 | Drinking surface water like rivers, streams and rain water reservoirs without any treatment as boiling or adding chlorine, is at high risk to cause food poisoning. |  |  |  |  |  |
| 15 | There is no risk of food poisoning from eating leftover cooked food kept in refrigerator for 2–3 days. |  |  |  |  |  |

**Third section: Food poisoning attitude.**

| **#** | **Statement # *** | **Strongly**  **agree** | **Agree** | **Uncertain** | **Disagree** | **Strongly**  **disagree** |
| --- | --- | --- | --- | --- | --- | --- |
| 1 | Raw milk is more healthy and nutritious than pasteurized or boiled milk. |  |  |  |  |  |
| 2 | There is no risk of disease from drinking raw goat or cow milk right after milking. |  |  |  |  |  |
| 3 | There is no risk of disease from drinking the milk of she camel right after milking. |  |  |  |  |  |
| 4 | Raw eggs are more healthy and nutritious than cooked ones. |  |  |  |  |  |
| 5 | There is no risk of disease from drinking raw eggs. |  |  |  |  |  |
| 6 | There is no risk of disease from eating raw meat of young animals. |  |  |  |  |  |
| 7 | Wiping vegetables or fruits make them safe to be eaten. |  |  |  |  |  |
| 8 | There is no risk of disease from eating cooked food kept at room temperature for one day if covered. |  |  |  |  |  |
| 9 | There is no risk of disease from eating unwashed vegetables and herbs picked up directly from the plant. |  |  |  |  |  |
| 10 | Baby feces is free from pathogenic microbes if he/she is not sick. |  |  |  |  |  |
| 11 | Rain water collected in reservoir is safe to drink without any treatment. |  |  |  |  |  |
| 12 | Food handlers without clinical symptoms, can contaminate food with pathogenic microbes which cause food poisoning. |  |  |  |  |  |
| 13 | Washing hands with soap and water prior to eating food is necessary to prevent food poisoning. |  |  |  |  |  |
| 14 | Thorough washing of vegetables and fruits in tap water is necessary to prevent food poisoning. |  |  |  |  |  |
| 15 | Washing hands with soap and water before preparing food is necessary to prevent food poisoning. |  |  |  |  |  |

**Fourth section: Food poisoning practices.**

| **#** | **Statement # *** | **Always** | **Often** | **Sometimes** | **Rarely** | **Never** |
| --- | --- | --- | --- | --- | --- | --- |
| 1 | Do you wash fresh vegetables and fruits in tap water before eating? |  |  |  |  |  |
| 2 | Do you wash your hands with soap and water before eating your meal? |  |  |  |  |  |
| 3 | Do you wash your hands with water and soap before preparing food? |  |  |  |  |  |
| 4 | Do you wash your hands with water and soap after handling raw unwashed vegetables? |  |  |  |  |  |
| 5 | Do you wash your hands with soap and water after using the toilet? |  |  |  |  |  |
| 6 | Do you wash your hands after contact with animals? |  |  |  |  |  |
| 7 | Do you eat fresh vegetables and fruits without washing? |  |  |  |  |  |
| 8 | Do you just wipe fresh vegetables and fruits before you eat them? |  |  |  |  |  |
| 9 | When you make a field trip, do you pick up vegetables or herbs from the plants and eat them without washing? |  |  |  |  |  |
| 10 | Do you eat raw eggs? |  |  |  |  |  |
| 11 | Do you eat half-cooked eggs (egg yolk is soft)? |  |  |  |  |  |
| 12 | Do you eat raw meat? |  |  |  |  |  |
| 13 | Do you eat half-cooked meat (inside is red)? |  |  |  |  |  |
| 14 | Do you drink raw cow or goat milk? |  |  |  |  |  |
| 15 | Do you drink raw milk of the camel? |  |  |  |  |  |
| 16 | Do you eat raw white cheese prepared from raw un-pasteurized milk? |  |  |  |  |  |
| 17 | Do you eat cooked food left at room temperature for over 6 h without sufficient heating? |  |  |  |  |  |
| 18 | Do you eat food from a restaurant/cafeteria looks not clean? |  |  |  |  |  |
| 19 | Do you drink from rainwater collected in reservoir or surface stream water without any treatment? |  |  |  |  |  |
| 20 | Do you eat food, like meat and rice and soup, by hand from a big bowl shared by several people? |  |  |  |  |  |

# These questions were adapted from Sharif L, Al-Malki T: **Knowledge, attitude and practice of Taif University students on food poisoning**. *Food Control* 2010, **21**(1):55-60.

* Permission to use this instrument to measure parents’ knowledge, attitude and practices regarding food poisoning in this study was obtained from the developers of the questionnaire Prof Labib Sharif
