# Supplementary material for: Knowledge, attitude and practices among parents regarding food poisoning: a cross-sectional study from Palestine
Source: BMC Public Health. 2019 May 16;19:586. doi: 10.1186/s12889-019-6955-2 (PMC6524328; doi:10.1186/s12889-019-6955-2)
Supplement: Supplementary file 2 — Table S1. Parents’ knowledge responses with correct answers. Table S2. Distribution of responses to each knowledge question with a five-point Likert scale ranked from 1 to 5 (Strongly disagree, disagree, not sure, agree, and strongly agree). Table S3. Parents’ attitude responses with correct answers. Table S4. Distribution of responses to each attitude question with a five-point Likert scale ranked from 1 to 5 (Strongly disagree, disagree, not sure, agree, and strongly agree). Table S5. Parents’ practice responses with correct answers. Table S6. Distribution of responses to each practice question with a five-point Likert scale ranked from 1 to 5 (Always yes, most of the time, sometimes, rarely, always no). (DOCX 27 kb) [file 12889_2019_6955_MOESM2_ESM.docx]

**Additional file 2: Table S1-S6.**  **Table S1:**Parents’ knowledge responses with correct answers. **Table S2:** Distribution of responses to each knowledge questionwith a five-point Likert scale ranked from 1 to 5 (Strongly disagree, disagree, not sure, agree, and strongly agree). **Table S3:** Parents’ attitude responses with correct answers. **Table S4:** Distribution of responses to each attitude questionwith a five-point Likert scale ranked from 1 to 5 (Strongly disagree, disagree, not sure, agree, and strongly agree). **Table S5:** Parents’ practice responses with correct answers. **Table S6:** Distribution of responses to each practice questionwith a five-point Likert scale ranked from 1 to 5 (Always yes, most of the time, sometimes, rarely, always no).

**Table S1:** Parents’ knowledge responses with correct answers

| **Question no.** | **Question statement** | **Agree (%)^*^** | **Not sure**  **(%)** | **Disagree (%)** |
| --- | --- | --- | --- | --- |
| **1** | “Food poisoning is caused by pathogenic microbes” (**appropriate**) | **364 (88.4)** | 29 (7.0) | 19 (4.6) |
| **2** | “Some toxins produced by microbes and cause food poisoning are resistant to heating temperature of food” (**appropriate**) | **202 (49.0)** | 123 (29.9) | 87 (21.1) |
| **3** | “Drinking raw milk is highly risky for food poisoning” (**appropriate**) | **359 (87.1)** | 25 (6.1) | 28 (6.8) |
| **4** | “Eating raw eggs is highly risky for food poisoning” (**appropriate**) | **292 (70.9)** | 63 (15.3) | 57 (13.8) |
| **5** | “Eating raw or half-cooked meat is highly risky for food poisoning” (**appropriate**) | **384 (93.2)** | 13 (3.2) | 15 (3.6) |
| **6** | “Eating raw unwashed vegetables is highly risky for food poisoning” (**appropriate**) | **377 (91.5)** | 15 (3.6) | 20 (4.9) |
| **7** | “Eating unwashed and not pealed fruits is highly risky for food poisoning “(**appropriate**) | **341 (82.8)** | 37 (8.9) | 34 (8.3) |
| **8** | “Food handlers with unhygienic practice could be the source of microbial  contamination of the food which causes food poisoning” (**appropriate**) | **342 (83.0)** | 36 (8.7) | 34 (8.3) |
| **9** | “Well cooked food is free from microbes which cause food poisoning” (**appropriate**) | **376 (91.3)** | 23 (5.6) | 13 (3.1) |
| **10** | “Eating uncovered leftover cooked food, kept at room temperature for 12–24 h, is at high risk to cause food poisoning” (**appropriate**) | **323 (78.3)** | 48 (11.7) | 41 (10.0) |
| **11** | “Raw white cheese processed from raw milk has a high risk of food poisoning” (**appropriate**) | **349 (84.7)** | 27 (6.6) | 36 (8.7) |
| **12** | “Pasteurized milk can be drunk directly with no risk of food poisoning” (**appropriate**) | **302 (73.3)** | 46 (11.2) | 64 (15.5) |
| **13** | “Keeping food at refrigerator temperature will slow down the microbial growth and multiplication, thus prevent food spoilage and food poisoning” (**appropriate**) | **372 (90.2)** | 20 (4.9) | 20 (4.9) |
| **14** | “Drinking surface water like rivers, streams and rain water reservoirs without any treatment as boiling or adding chlorine, is at high risk to cause food poisoning” (**appropriate**) | **306 (74.3)** | 47 (11.4) | 59 (14.3) |
| **15** | There is no risk of food poisoning from eating leftover cooked food kept in refrigerator for 2–3 days (**appropriate**) | **245 (59.5)** | 63 (15.3) | 104 (25.2) |

*correct answers are in bold font.

**Table S2:** Distribution of responses to each knowledge questionwith a five-point Likert scale ranked from 1 to 5 (Strongly disagree, disagree, Not sure, agree, and strongly agree)

| **Question no.** | **Strongly agree (%)** | **Agree (%)** | **Not sure (%)** | **Disagree (%)** | **Strongly disagree (%)** |
| --- | --- | --- | --- | --- | --- |
| **1** | 208 (50.5) | 156 (37.9) | 29 (7.0) | 16 (3.9) | 3 (0.7) |
| **2** | 86 (20.9) | 116 (28.2) | 123 (29.9) | 62 (15.0) | 25 (6.1) |
| **3** | 251 (60.9) | 108 (26.2) | 25 (6.1) | 18 (4.4) | 10 (2.4) |
| **4** | 181 (43.9) | 111 (26.9) | 63 (15.3) | 43 (10.4) | 14 (3.4) |
| **5** | 303 (73.5) | 81 (19.7) | 13 (3.2) | 9 (2.2) | 6 (1.5) |
| **6** | 232 (56.3) | 145 (35.2) | 15 (3.6) | 13 (3.2) | 7 (1.7) |
| **7** | 186 (45.1) | 155 (37.6) | 37 (9.0) | 28 (6.8) | 6 (1.5) |
| **8** | 190 (46.1) | 152 (36.9) | 36 (8.7) | 26 (6.3) | 8 (1.9) |
| **9** | 274 (66.5) | 102 (24.8) | 23 (5.6) | 11 (2.7) | 2 (0.5) |
| **10** | 210 (51.0) | 113 (27.4) | 48 (11.7) | 34 (8.3) | 7 (1.7) |
| **11** | 230 (55.8) | 119 (28.9) | 27 (6.6) | 25 (6.1) | 11 (2.7) |
| **12** | 152 (36.9) | 150 (36.4) | 46 (11.2) | 49 (11.9) | 15 (3.6) |
| **13** | 254 (61.7) | 118 (28.6) | 20 (4.9) | 14 (3.4) | 6 (1.5) |
| **14** | 206 (50.0) | 100 (24.3) | 47 (11.4) | 45 (10.9) | 14 (3.4) |
| **15** | 128 (31.1) | 117 (28.4) | 63 (15.3) | 48 (11.7) | 56 (13.6) |

**Table S3:** Parents’ attitude responses with correct answers

| **Question no.** | **Question statement** | **Agree (%)*** | **Disagree (%)*** |
| --- | --- | --- | --- |
| **1** | “Raw milk is more healthy and nutritiousthan pasteurized or boiled milk” (**inappropriate**) | 96 (23.3) | **316 (76.7)** |
| **2** | “There is no risk of disease from drinking raw goat or cow milk right after milking” (**inappropriate**) | 74 (18.0) | **338 (82.0)** |
| **3** | “There is no risk of disease from drinking the milk of she camel right after milking” (**inappropriate**) | 118 (28.6) | **294 (71.4)** |
| **4** | “Raw eggs are more healthy and nutritious than cooked ones” (**inappropriate**) | 130 (31.6) | **282 (68.4)** |
| **5** | “There is no risk of disease from drinking raw eggs” (**inappropriate**) | 149 (36.2) | **263 (63.8)** |
| **6** | “There is no risk of disease from eating raw meat of young animals” (**inappropriate**) | 44 (10.7) | **368 (89.3)** |
| **7** | “Wiping vegetables or fruits make them safe to be eaten” (**inappropriate**) | 71 (17.2) | **341 (82.8)** |
| **8** | “There is no risk of disease from eating cooked food kept at room temperature for one day if covered” (**inappropriate**) | 180 (43.7) | **232 (56.3)** |
| **9** | “There is no risk of disease from eating unwashed vegetables and herbs picked up directly from the plant” (**inappropriate**) | 83 (20.1) | **329 (79.9)** |
| **10** | “Baby feces is free from pathogenic microbes if he/she is not sick” (**inappropriate**) | 249 (60.4) | **163 (39.6)** |
| **11** | “Rain water collected in reservoir is safe to drink without any treatment” (**inappropriate**) | 190 (46.1) | **222 (53.9)** |
| **12** | “Food handlers without clinical symptoms, can contaminate food with pathogenic microbes which cause food poisoning” (**appropriate**) | **249 (60.4)** | 163 (39.6) |
| **13** | “Washing hands with soap and water prior to eating food is necessary to prevent food poisoning” (**appropriate**) | **396 (96.1)** | 16 (3.9) |
| **14** | “Thorough washing of vegetables and fruits in tap water is necessary to prevent food poisoning” (**appropriate**) | **394 (95.6)** | 18 (4.4) |
| **15** | “Washing hands with soap and water before preparing food is necessary to prevent food poisoning” (**appropriate**) | **397 (96.4)** | 15 (3.6) |

*correct answers are in bold font

**Table S4:** Distribution of responses to each attitude questionwith a five-point Likert scale ranked from 1 to 5 (Strongly disagree, disagree, not sure, agree, and strongly agree)

| **Question no.** | **Strongly agree (%)** | **Agree (%)** | **Not sure (%)** | **Disagree (%)** | **Strongly disagree (%)** |
| --- | --- | --- | --- | --- | --- |
| **1** | 28 (6.8) | 37 (9.0) | 31 (7.5) | 123 (29.9) | 193 (46.8) |
| **2** | 18 (4.4) | 24 (5.8) | 32 (7.8) | 104 (25.2) | 234 (56.8) |
| **3** | 21 (5.1) | 20 (4.9) | 77 (18.7) | 88 (21.4) | 206 (50.0) |
| **4** | 30 (7.3) | 22 (5.3) | 78 (18.9) | 130 (31.6) | 152 (36.9) |
| **5** | 33 (8.0) | 49 (11.9) | 67 (16.3) | 129 (31.3) | 134 (32.5) |
| **6** | 12 (2.9) | 8 (1.9) | 24 (5.8) | 121 (29.4) | 247 (60.0) |
| **7** | 9 (2.2) | 20 (4.9) | 42 (10.2) | 173 (42.0) | 168 (40.8) |
| **8** | 37 (9.0) | 91 (22.1) | 52 (12.6) | 121 (29.4) | 111 (26.9) |
| **9** | 12 (2.9) | 36 (8.7) | 35 (8.5) | 168 (40.8) | 161 (39.1) |
| **10** | 50 (12.1) | 56 (13.6) | 143 (34.7) | 89 (21.6) | 74 (18.0) |
| **11** | 42 (10.2) | 63 (15.3) | 85 (20.6) | 120 (29.1) | 102 (24.8) |
| **12** | 132 (32.0) | 117 (28.4) | 80 (19.4) | 41 (10.0) | 42 (10.2) |
| **13** | 332 (80.6) | 64 (15.5) | 4 (1.0) | 5 (1.2) | 7 (1.7) |
| **14** | 303 (73.5) | 91 (22.1) | 6 (1.5) | 7 (1.7) | 5 (1.2) |
| **15** | 328 (79.6) | 69 (16.7) | 5 (1.2) | 2 (0.5) | 8 (1.9) |

**Table S5:** Parents’ practice responses with correct answers

| **Question no.** | **Question statement** | **Yes (%)*** | **No (%)*** |
| --- | --- | --- | --- |
| **1** | “Do you wash fresh vegetables and fruits in tap water before eating?” (**appropriate)** | **404 (98.1)** | 8 (1.9) |
| **2** | “Do you wash your hands with soap and water before eating your meal?” (**appropriate)** | **395 (95.9)** | 17 (4.1) |
| **3** | “Do you wash your hands with water and soap before preparing food?” (**appropriate)** | **395 (95.9)** | 17 (4.1) |
| **4** | “Do you wash your hands with water and soap after handling raw unwashed vegetables?” (**appropriate)** | **298 (72.3)** | 114 (27.7) |
| **5** | “Do you wash your hands with soap and water after using the toilet?” (**appropriate)** | **409 (99.3)** | 3 (0.7) |
| **6** | “Do you wash your hands after contact with animals?” (**appropriate**) | **405 (98.3)** | 7 (1.7) |
| **7** | “Do you eat fresh vegetables and fruits without washing?” (**inappropriate)** | 131 (31.8) | **281 (68.2)** |
| **8** | “Do you just wipe fresh vegetables and fruits before you eat them?” (**inappropriate)** | 148 (35.9) | **264 (64.1)** |
| **9** | “When you make a field trip, do you pick up vegetables or herbs from the plants and eat them without washing?” (**inappropriate)** | 143 (34.7) | **269 (65.3)** |
| **10** | “Do you eat raw eggs?” (**inappropriate)** | 25 (6.1) | **387 (93.9)** |
| **11** | “Do you eat half-cooked eggs (egg yolk is soft)?” (**inappropriate)** | 102 (24.8) | **310 (75.2)** |
| **12** | “Do you eat raw meat?” (**inappropriate)** | 16 (3.9) | **396 (96.1)** |
| **13** | “Do you eat half-cooked meat (inside is red)?” (**inappropriate)** | 24 (5.8) | **388 (94.2)** |
| **14** | “Do you drink raw cow or goat milk?” (**inappropriate)** | 24 (5.8) | **388 (94.2)** |
| **15** | “Do you drink raw milk of the camel?” (**inappropriate)** | 23 (5.6) | **389 (94.4)** |
| **16** | “Do you eat raw white cheese prepared from raw un-pasteurized milk?” (**inappropriate)** | 83 (20.1) | **329 (79.9)** |
| **17** | “Do you eat cooked food left at room temperature for over 6 h without sufficient heating?” (**inappropriate)** | 116 (28.2) | **296 (71.8)** |
| **18** | “Do you eat food from a restaurant/cafeteria looks not clean?” (**inappropriate)** | 15 (3.6) | **397 (96.4)** |
| **19** | “Do you drink from rainwater collected in reservoir or surface stream water without any treatment?” (**inappropriate)** | 69 (16.7) | **343 (83.3)** |
| **20** | “Do you eat food, like meat and rice and soup, by hand from a big bowl shared by several people?” (**inappropriate)** | 34 (8.3) | **378 (91.7)** |

*correct answers are in bold

**Table S6:** Distribution of responses to each practice questionwith a five-point Likert scale ranked from 1 to 5 (Always yes, most of the time, sometimes, rarely, always no)

| **Question no.** | **Always yes (%)** | **Most of the time (%)** | **Sometimes (%)** | **Rarely (%)** | **Always no (%)** |
| --- | --- | --- | --- | --- | --- |
| **1** | 363 (88.1) | 41 (10.0) | 6 (1.5) | 1(0.2) | 1 (0.2) |
| **2** | 353 (85.7) | 42 (10.2) | 15 (3.6) | 2 (0.5) | 0 (0.0) |
| **3** | 358 (86.9) | 37 (9.0) | 12 (2.9) | 2 (0.5) | 3 (0.7) |
| **4** | 232 (56.3) | 66 (16.0) | 46 (11.2) | 33 (8.0) | 35 (8.5) |
| **5** | 406 (98.5) | 3 (0.7) | 3 (0.7) | 0 (0.0) | 0 (0.0) |
| **6** | 395 (95.9) | 10 (2.4) | 2 (0.5) | 3 (0.7) | 2 (0.5) |
| **7** | 19 (4.6) | 33 (8.0) | 79 (19.2) | 64 (15.5) | 217 (52.7) |
| **8** | 19 (4.6) | 25 (6.1) | 104 (25.2) | 63 (15.3) | 201 (48.8) |
| **9** | 26 (6.3) | 32 (7.8) | 85 (20.6) | 75 (18.2) | 194 (47.1) |
| **11** | 8 (1.9) | 6 (1.5) | 11 (2.7) | 25 (6.1) | 362 (87.9) |
| **11** | 25 (6.1) | 26 (6.3) | 51 (12.4) | 45 (10.9) | 265 (64.3) |
| **12** | 3 (0.7) | 6 (1.5) | 7 (1.7) | 8 (1.9) | 388 (94.2) |
| **13** | 6 (1.5) | 4 (1.0) | 14 (3.4) | 17 (4.1) | 371 (90.0) |
| **14** | 9 (2.2) | 6 (1.5) | 9 (2.2) | 12 (2.9) | 376 (91.3) |
| **15** | 9 (2.2) | 6 (1.5) | 8 (1.9) | 14 (3.4) | 375 (91.0) |
| **16** | 13 (3.2) | 16 (3.9) | 54 (13.1) | 42 (10.2) | 287 (69.7) |
| **17** | 21 (5.1) | 11 (2.7) | 84 (20.4) | 58 (14.1) | 238 (57.8) |
| **18** | 9 (2.2) | 2 (0.5) | 4 (1.0) | 7 (1.7) | 390 (94.7) |
| **19** | 24 (5.8) | 23 (5.6) | 22 (5.3) | 40 (9.7) | 303 (73.5) |
| **20** | 17 (4.1) | 8 (1.9) | 9 (2.2) | 22 (5.3) | 356 (86.4) |
